# CRISPR/Cas9-Based Knockout of GNAQ Reveals Differences in Host Cell Signaling Necessary for Egress of Apicomplexan Parasites

**DOI:** 10.1128/mSphere.01001-20

**Published:** 2020-12-23

**Authors:** Paul-Christian Burda, Hugo Bisio, Jean-Baptiste Marq, Dominique Soldati-Favre, Volker T. Heussler

**Affiliations:** a Institute of Cell Biology, University of Bern, Bern, Switzerland; b University of Geneva, Geneva, Switzerland; University at Buffalo

**Keywords:** GNAQ, *Plasmodium*, *Toxoplasma*, egress, host cell signaling, malaria

## Abstract

Toxoplasma gondii and members of the genus *Plasmodium* are obligate intracellular parasites that leave their infected host cell upon a tightly controlled process of egress. Intracellular replication of the parasites occurs within a parasitophorous vacuole, and its membrane as well as the host plasma membrane need to be disrupted during egress, leading to host cell lysis. While several parasite-derived factors governing egress have been identified, much less is known about host cell factors involved in this process. Previously, RNA interference (RNAi)-based knockdown and antibody-mediated depletion identified a host signaling cascade dependent on guanine nucleotide-binding protein subunit alpha q (GNAQ) to be required for the egress of *Toxoplasma* tachyzoites and *Plasmodium* blood stage merozoites. Here, we used CRISPR/Cas9 technology to generate HeLa cells deficient in GNAQ and tested their capacity to support the egress of T. gondii tachyzoites and Plasmodium berghei liver stage parasites. While we were able to confirm the importance of GNAQ for the egress of T. gondii, we found that the egress of P. berghei liver stages was unaffected in the absence of GNAQ. These results may reflect differences between the lytic egress process in apicomplexans and the formation of host cell-derived vesicles termed merosomes by P. berghei liver stages.

**IMPORTANCE** The coordinated release of apicomplexan parasites from infected host cells prior to reinvasion is a critical process for parasite survival and the spread of infection. While *Toxoplasma* tachyzoites and *Plasmodium* blood stages induce a fast disruption of their surrounding membranes during their egress from host cells, *Plasmodium* liver stages keep the host cell membrane intact and leave their host cell in host cell-derived vesicles called merosomes. The knockout of GNAQ, a protein involved in G-protein-coupled receptor signaling, demonstrates the importance of this host factor for the lytic egress of T. gondii tachyzoites. Contrastingly, the egress of P. berghei is independent of GNAQ at the liver stage, indicating the existence of a mechanistically distinct strategy to exit the host cell.

## Abstract (precis)

The coordinated release of apicomplexan parasites from infected host cells prior to reinvasion is a critical process for parasite survival and the spread of infection. While *Toxoplasma* tachyzoites and *Plasmodium* blood stages induce a fast disruption of their surrounding membranes during their egress from host cells, *Plasmodium* liver stages keep the host cell membrane intact and leave their host cell in host cell-derived vesicles called merosomes.

## OBSERVATION

Apicomplexan parasites, including Toxoplasma gondii and Plasmodium falciparum, cause significant morbidity and mortality worldwide. While T. gondii can replicate in virtually any nucleated cell in a wide range of warm-blooded vertebrate hosts, P. falciparum multiplies only within hepatocytes and red blood cells in the human host. In their host cells, parasites are contained in a parasitophorous vacuole (PV) that is surrounded by the PV membrane (PVM). Parasites must escape from the PV and the host cell prior to invading other cells and spreading the infection.

In the case of T. gondii tachyzoites and *Plasmodium* blood stage merozoites, this egress process is a rapid event, whereby first the PVM is disrupted and seconds to minutes later the host cell plasma membrane (HCM) is also disrupted (1–3). During *Plasmodium* release from hepatocytes, the vacuole is similarly ruptured; however, the HCM stays intact for several hours, enabling the formation of host cell-derived vesicles termed merosomes that transport parasites from the liver to the bloodstream. Only then does the HCM-derived merosomal membrane rupture, whereby hepatic merozoites are released to infect erythrocytes (4, 5).

Egress is a highly regulated process, and several parasite proteins involved have been identified in *Toxoplasma* and *Plasmodium*, including proteases, kinases, pore-forming proteins, as well as phospholipases (reviewed in references 6 and 7). Much less is known about the contribution of host cell factors to this process. However, RNA interference (RNAi)-based knockdown and antibody-mediated depletion previously showed that a host signaling cascade dependent on guanine nucleotide-binding protein subunit alpha q (GNAQ) is necessary for the egress of T. gondii tachyzoites and P. falciparum blood stage parasites from host cells. In this cascade, putative parasite G-protein-coupled receptor (GPCR) ligands overstimulate host GPCRs, leading to the GNAQ-mediated activation of protein kinase C, which weakens the host cell cytoskeleton by the phosphorylation of adducin. This in turn induces calcium influx through the mechanosensitive cation channel TRPC6 that activates host calpain-1 to proteolyze the host cytoskeleton, enabling parasite release (8, 9).

### CRISPR/Cas9-based knockout of GNAQ.

**FIG 1:**
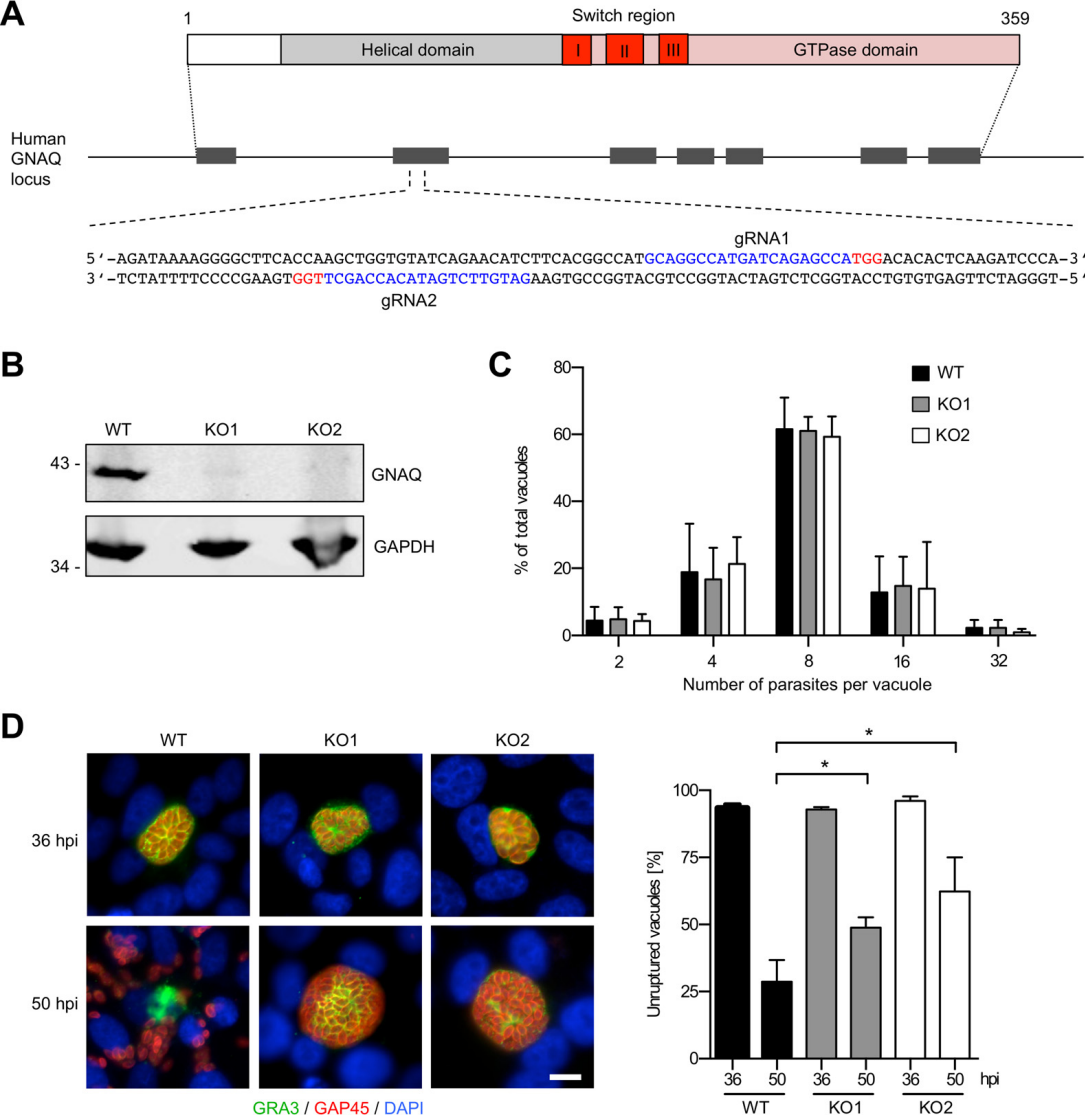
Egress of T. gondii depends on host cell GNAQ. (A) Schematic diagram of the functional domains of the GNAQ protein and localization of CRISPR gRNA target sites in exon 2 of the *GNAQ* locus (blue) and protospacer adjacent motif (PAM) sequences (red). (B) Western blotting of the WT and the two KO cell lines confirms the successful deletion of GNAQ. GAPDH served as a loading control. (C) T. gondii parasites show similar growth in WT and GNAQ-KO cells. The number of parasites per vacuole was determined at 24 hpi. (D) Natural egress of T. gondii is impaired in GNAQ-KO cells. For the determination of natural egress rates, the percentage of unruptured vacuoles was determined at 36 and 50 hpi. Images from representative immunofluorescence analyses of parasites with unruptured and ruptured PVM are shown on the left. The PVM marker GRA3 is displayed in green, while the parasite periphery marker GAP45 is shown in red. Nuclei were visualized with DAPI (blue). Bar, 10 μm. For panels C and D, means ± standard deviations (SD) from three independent experiments are shown. In each of these experiments, at least 100 vacuoles per time point were counted. For statistical evaluation of the growth and egress of parasites within GNAQ-KO cells in comparison to WT cells, one-way analysis of variance (ANOVA) followed by a Holm-Sidak multiple-comparison test was performed. All statistically significant differences are indicated (*, *P* < 0.05).

We aimed at testing whether GNAQ-mediated host cell signaling also contributes to *Plasmodium* liver stage egress and therefore used CRISPR/Cas9 technology to generate GNAQ-deficient knockout (KO) cells. In order to minimize the risk of off-target mutations, we applied the CRISPR/Cas9 paired nickase approach, which leads to two single-strand nicks situated close to each other on the genomic DNA (10). Two guide RNAs (gRNAs) targeting GNAQ in exon 2 were designed (Fig. 1A) and expressed together with Cas9 in HeLa cells. These cells were chosen because they fully support T. gondii tachyzoite as well as Plasmodium berghei liver stage development (11, 12) and are particularly well suited for imaging-based analysis of liver stage egress (13). Two single clonal cell lines (named KO1 and KO2) were obtained, and the absence of GNAQ was confirmed by Western blotting (Fig. 1B). Furthermore, successful manipulation of the *GNAQ* locus was confirmed in these two clones by genomic PCR followed by sequencing of the mutated region in exon 2 (see Fig. S1 in the supplemental material).

10.1128/mSphere.01001-20.1 FIG S1 Genotyping results for GNAQ-KO cell lines. Download FIG S1, TIF file, 0.7 MB. Copyright © 2020 Burda et al. 2020 Burda et al. This content is distributed under the terms of the Creative Commons Attribution 4.0 International license.

### GNAQ-mediated signaling is involved in the egress of T. gondii.

We next infected wild-type (WT) and GNAQ-KO cells with T. gondii tachyzoites. Upon the fixation of infected cells at 24 h postinfection (hpi), counting of the number of parasites per vacuole showed, as expected, that GNAQ-KO cells fully supported T. gondii growth compared to WT cells (Fig. 1C). Interestingly, the determination of the rate of natural egress by fixation and counting of infected cells at 36 and 50 hpi revealed a statistically significantly higher percentage of unruptured vacuoles in the two GNAQ-KO lines than in WT cells (Fig. 1D). These findings confirmed previous RNAi-based results showing that GNAQ-mediated signaling is important for the egress of T. gondii (8).

### P. berghei liver stage egress occurs independently of GNAQ.

**FIG 2:**
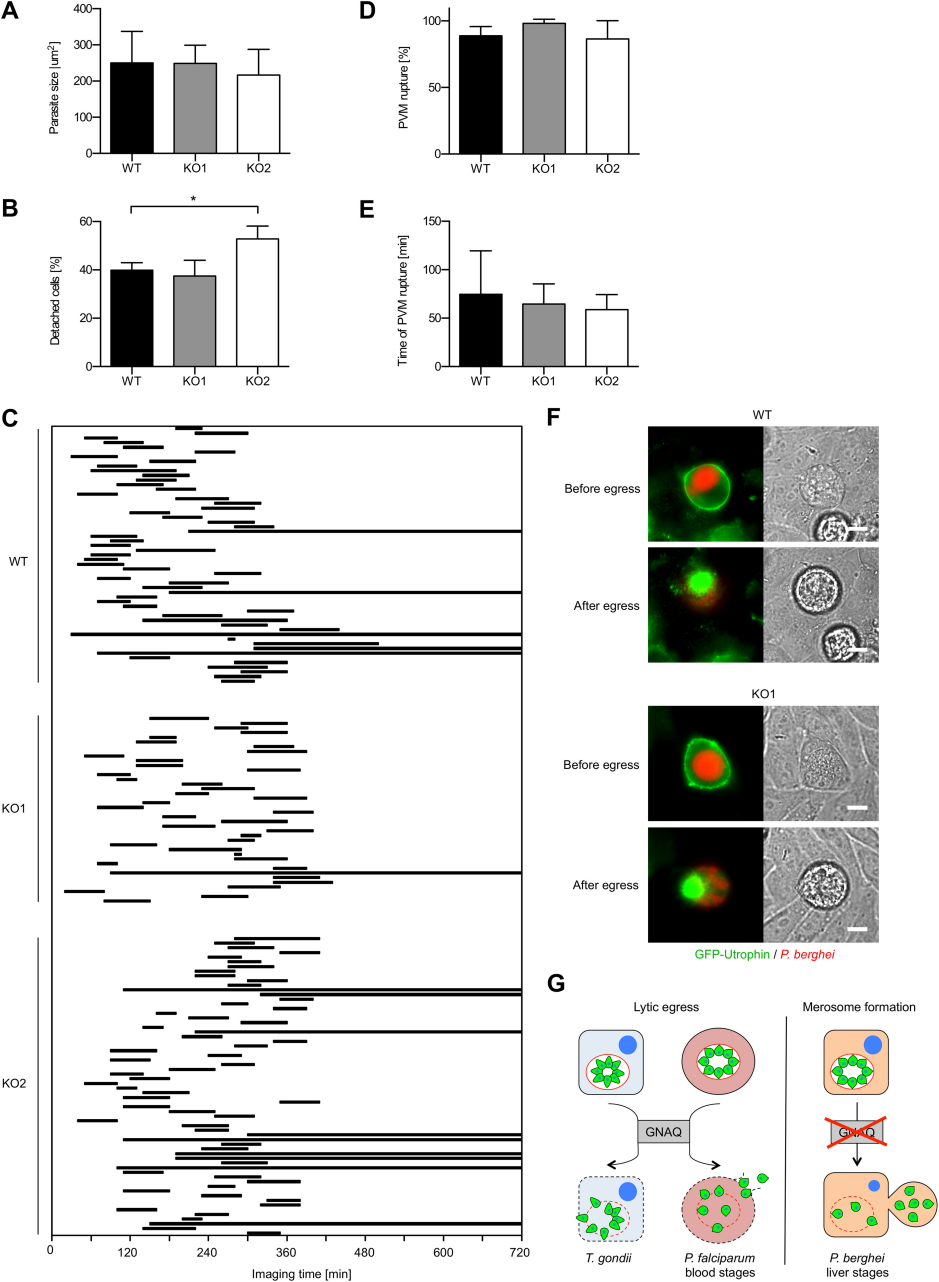
P. berghei liver stage egress is independent of host cell GNAQ. (A) P. berghei parasites show comparable growth in WT and GNAQ-KO cells. WT and GNAQ-KO cells were infected with P. berghei parasites, and at 48 hpi, the average parasite size (area) was determined by density slicing using ImageJ from 50 to 100 parasites per experiment. Shown are means ± SD from five independent experiments. (B) The formation of detached cells is not impaired in GNAQ-deficient host cells. Detached cells in the supernatant were counted at 65 hpi and normalized to the number of infected cells at 48 hpi. Shown are means ± SD from five independent experiments, each performed in triplicate. (C to E) P. berghei liver stage PVM rupture is not affected by the absence of GNAQ. WT and GNAQ-KO cells were infected with sporozoites, and the percentage of merozoite-forming parasites that ruptured the PVM and the time difference between the successful formation of merozoites and PVM rupture were measured by quantitative live-cell imaging. The influx of GFP into the PV was used as a measure of PVM rupture. Imaging was started at ∼55 hpi and lasted for 12 h. (C) Time between the formation of merozoites and PVM rupture. Each line represents the time difference between successful merozoite formation (beginning of the line) and PVM rupture (end of the line) and corresponds to one analyzed parasite. Continuous lines indicate parasites that did not rupture the PVM at all, which were not considered for the determination of the average PVM rupture time in panel E. Shown are combined data from three independent imaging experiments per cell line. (D) Percentage of merozoite-forming parasites that ruptured the PVM. The percentage of PVM rupture was determined in each of the three independent imaging experiments, in which the number of parasites that successfully developed to merozoites within the first 6 h of imaging was set to 100% in each experiment. Based on these, the percentage of parasites that successfully ruptured the PVM was calculated. Shown are means ± SD. (E) Elapsed time from merozoite formation to PVM rupture determined for all parasites that ruptured the PVM. Displayed are means ± SD. For all statistical analyses between WT and GNAQ-KO cells, one-way ANOVA followed by a Holm-Sidak multiple-comparison test was performed. All statistically significant differences are indicated (*, *P* < 0.05). (F) Egress-associated breakdown of the host actin cytoskeleton is comparable in WT and GNAQ-deficient cells. Cells expressing the filamentous actin marker GFP-utrophin (green) were infected with P. berghei parasites constitutively expressing mCherry (red), and their egress was studied by live-cell time-lapse microscopy. Representative images before and after egress are shown. Bars, 10 μm. See also Movies S1 and S2 in the supplemental material. (G) Comparison and GNAQ dependence of the egress strategies of T. gondii tachyzoites and P. falciparum blood stages versus P. berghei liver stages. The PVM is displayed in red.

To study *Plasmodium* liver stage development in these cells, we infected WT and GNAQ-KO cells with transgenic, fluorescent P. berghei sporozoites and measured parasite size at 48 hpi. No change of size was observed (Fig. 2A), confirming that GNAQ deficiency does not affect the growth of liver stage parasites up to this time point.

10.1128/mSphere.01001-20.2 MOVIE S1 Host cell actin modulation during P. berghei liver stage egress in WT cells (related to Fig. 2). WT cells expressing GFP-utrophin (green) were infected with mCherry-expressing parasites (red). Parasite development was monitored by epifluorescence live-cell time-lapse microscopy, and imaging was started at around 55 hpi. The movie was acquired with a 10-min time interval between frames and is shown at 4 frames per s. Hours and minutes from the start of the movie are displayed. Bar, 10 μm. Download Movie S1, AVI file, 2.0 MB. Copyright © 2020 Burda et al. 2020 Burda et al. This content is distributed under the terms of the Creative Commons Attribution 4.0 International license.

10.1128/mSphere.01001-20.3 MOVIE S2 Host cell actin modulation during P. berghei liver stage egress in GNAQ-KO cells (related to Fig. 2). KO1 cells expressing GFP-utrophin (green) were infected with mCherry-expressing parasites (red). Parasite development was monitored by epifluorescence live-cell time-lapse microscopy, and imaging was started at around 55 hpi. The movie was acquired with a 10-min time interval between frames and is shown at 4 frames per s. Hours and minutes from the start of the movie are displayed. Bar, 10 μm. Download Movie S2, AVI file, 1.4 MB. Copyright © 2020 Burda et al. 2020 Burda et al. This content is distributed under the terms of the Creative Commons Attribution 4.0 International license.

Upon the formation of merozoites, parasites rupture the PVM, leading to their release into the host cell cytoplasm and the detachment of the infected host cell, which typically occurs at between 55 and 60 hpi (4, 14). Since *in vitro* cell detachment is considered a robust marker for liver stage egress, we next counted the number of detached cells. Remarkably, no major difference between WT and GNAQ-KO cells was observed (Fig. 2B), suggesting that, in contrast to T. gondii tachyzoites, P. berghei liver stage egress is independent of host cell GNAQ. The formation of detached cells was even greater in KO2 cells than in WT cells, which is presumably derived from a clonal effect and not related to the loss of GNAQ, as it was observed in only one of the two KO cell lines. To study P. berghei liver stage egress in these cells in more detail, we used a previously established assay based on quantitative live-cell time-lapse microscopy that analyzes PVM rupture (14). Differences in neither the percentage of parasites undergoing PVM rupture nor the timing of this process were visible (Fig. 2C to E), further supporting the notion that P. berghei liver stage exit does not depend on GNAQ.

Of relevance, P. berghei liver stages induce a breakdown of the host cell actin cytoskeleton during their release from host cells (13). We thus expressed the filamentous actin marker green fluorescent protein (GFP)-utrophin (15) in WT and GNAQ-KO cells and analyzed the egress of liver stages in these cells by live-cell time-lapse microscopy. In line with data from the other egress assays, the actin cytoskeleton also collapsed during parasite egress from GNAQ-deficient cells, similar to the observations in WT cells (Fig. 2F; Movies S1 and S2), indicating that GNAQ KO also does not affect the remodeling of the host actin cytoskeleton.

### Discussion.

After the rupture of the PVM, liver stage-derived *Plasmodium* merozoites leave the infected host cells packed in merosomes. The HCM-derived merosomal membrane needs to remain intact for several hours until merosomes have reached the bloodstream. At this point, merozoites are released to infect erythrocytes, thereby initiating the pathogenic blood phase of infection (4, 5). This strategy contrasts with the lytic egress of T. gondii tachyzoites and *Plasmodium* blood stage merozoites, whereby the PVM and HCM are disrupted in quick succession, leading to fast parasite exit and reinvasion of neighboring cells (1–3). In this study, we provide evidence that differences in GNAQ-mediated host cell signaling between these two different egress strategies might exist (Fig. 2G). We observed an increased percentage of T. gondii tachyzoite stage parasites that did not egress from their PV in GNAQ-KO cells in comparison to WT cells, while PV rupture and the subsequent formation of detached cells of P. berghei liver stages were not affected by KO of GNAQ. Of note, the T. gondii egress phenotype in our GNAQ-deficient HeLa cells was not as strong as that previously reported for U2OS cells, in which GNAQ was knocked down by RNAi (8). This could be due to either differences in the host cell type or the fact that KO cells generated by CRISPR/Cas9 have more time to upregulate compensating factors than RNAi-based knockdown cells, and this might well affect the observed phenotype. Importantly, natural egress in T. gondii is triggered by a combination of factors derived from the parasite (i.e., diacylglycerol kinase 2 activity) as well as from the host (i.e., host calcium raise or potassium drop) (7, 16). Due to this redundancy, the absence of one important host cell factor is more likely to affect egress efficiency rather than to completely abolish it (7).

Liver stage egress consists of PVM rupture and the induction of merosome formation and is ultimately terminated by the rupture of merosomes in the bloodstream, leading to merozoite release (4). While PVM rupture and merosome formation appear to be unaffected by KO of GNAQ, the potential importance of GNAQ in merosome rupture currently cannot be excluded and needs further investigation.

P. falciparum merozoites egress spontaneously from red blood cells in *in vitro* cultures, whereas P. berghei merozoites are not able to do so (17, 18). This, together with some transcriptional variation (19), points to certain differences in the egress process between both species. Thus, for a complete understanding of GNAQ function during apicomplexan release and to see if the differences in GNAQ dependence are a general feature of *Plasmodium* liver stage egress, it remains to be shown if the observations made here on P. berghei liver stages are also true for other *Plasmodium* species, including P. falciparum.

While the kinetics of egress are to some extent driven by parasite effector proteins that differ between species and stages, our study indicates that differences in host cell signaling might also be important. Future studies are necessary to work out the molecular basis of these differences and if other host cell factors differentially regulate the egress of apicomplexan parasites.

### Methods.

**(i) Ethics statement.** All experiments were conducted in strict accordance with the guidelines of the Swiss Tierschutzgesetz (TSchG) (Animal Rights Laws) and approved by the ethical committee of the University of Bern (permit number BE109/13).

**(ii) Experimental animals.** Mice used in the experiments were between 6 and 10 weeks of age. BALB/c mice were obtained from Harlan Laboratories or bred in the central animal facility of the University of Bern. Mosquito feeds were performed on mice anesthetized with ketamine hydrochloride-medetomidine hydrochloride (Ketavet-Domitor; Pfizer), and all efforts were made to minimize suffering.

**(iii) Parasite lines.** P. berghei ANKA parasites constitutively expressing mCherry in the parasite cytosol (14) and T. gondii RH-ΔHGPRT and Δku80 (20) tachyzoites were used for the experiments.

**(iv) HeLa cell cultivation, transfection, and P. berghei infection.** HeLa cells (a gift from Robert Menard, Pasteur Institute, Paris, France) were authenticated by short tandem repeat (STR) DNA profiling (Microsynth) and cultured as described previously (12). A total of 2 × 10^6^ HeLa cells were transfected with 4 μg of pEGFP-N3 (Clontech) or GFP-utrophin (15) (Addgene plasmid 26737) plasmid DNA using program T-28 in an Amaxa Nucleofector (Lonza). Cells were subsequently seeded into glass-bottom dishes (MatTek, In Vitro Scientific) and infected the next day with P. berghei sporozoites as previously described (14).

**(v) Generation and confirmation of GNAQ-deficient HeLa cells.** The CRISPR/Cas9 nickase system described previously (10) was used to knock out GNAQ in HeLa cells. CRISPR gRNA pairs were designed and selected using the Benchling CRISPR gRNA design tool to target exon 2 of the *GNAQ* gene. Cloning of the 2 gRNA oligonucleotides GCAGGCCATGATCAGAGCCA and GATGTTCTGATACACCAGCT into the plasmid pX335-U6-Chimeric_BB-CBh-hSpCas9n(D10A) (Addgene plasmid 42335, supplied by Feng Zhang) was performed according to the protocol of the Zhang laboratory (21). HeLa cells were transfected with 5 μg of the two pX335 plasmids, each encoding one gRNA sequence. Three days after transfection, transfected cells were plated into 96-well plates to obtain clonal cell lines. Single colonies were expanded and screened for the absence of GNAQ by Western blot analysis. Two clones that showed no GNAQ signal were further analyzed on the genomic DNA level. Genomic DNA was isolated from these cells using QuickExtract DNA extraction solution 1.0 (Epicentre), and the regions of interest were amplified by PCR using primers GNAQ-Ex2-fw (AGACTGAGATGGCACTGTGG) and GNAQ-Ex2-rev (TTGCTGAACATCCAAATATGCC). PCR products were subcloned into pJet1.2 (Thermo Fisher Scientific), and 15 plasmids from each cell line were sequenced.

**(vi) SDS-PAGE and Western blot analysis.** A total of 0.25 × 10^6^ HeLa cells were transferred to an Eppendorf tube and spun down in a tabletop centrifuge. The supernatant was removed, and cells were resuspended in 40 μl of water. Ten microliters of 5× SDS sample buffer was added, and after vortexing, the samples were heated at 95°C for 5 min. After cooling down, 1 μl of Benzonase per tube was added, and the samples were incubated for another 5 min at 37°C. Samples were then resolved by SDS-PAGE and transferred to nitrocellulose membranes (LI-COR). The membranes were blocked in 5% milk in Tris-buffered saline–Tween (TBS-T), followed by incubation with the following primary antibodies that were diluted in TBS-T containing 5% milk: rabbit anti-GNAQ (catalog number sc-393; Santa Cruz) (1:1,000) and chicken anti-glyceraldehyde-3-phosphate dehydrogenase (GAPDH) (catalog number AB2302; Millipore) (1:5,000). After three washes in TBS-T, membranes were incubated with the following similarly diluted secondary antibodies: goat anti-mouse-800CW (LI-COR) (1:10,000) and goat anti-chicken-680RD (LI-COR) (1:10,000). Subsequently, membranes were washed another three times with TBS-T and scanned on a LI-COR Odyssey imaging system.

**(vii) Analysis of T. gondii development and egress.** T. gondii tachyzoites were grown in human foreskin fibroblast (ATCC CRL-1634) monolayers in Dulbecco’s modified Eagle’s medium (Gibco) supplemented with 5% fetal calf serum, 2 mM glutamine, and 25 μg/ml gentamicin (37°C with 5% CO_2_). As previously described (16), for the study of parasite development and natural egress, 1 × 10^5^ HeLa cells were plated in 24-well plates and allowed to attach overnight. A total of 1 × 10^5^ freshly egressed RH parasites were allowed to invade for 3 to 5 min before extensive washing in order to obtain partially synchronous cultures as previously described (22). Intracellular parasite growth was measured at 24 hpi. Natural egress of the parasites was monitored at 36 and 50 hpi by immunofluorescence analysis.

**(viii) Immunofluorescence analysis.** T. gondii-infected cells on coverslips were fixed with 4% paraformaldehyde (PFA)–0.05% glutaraldehyde (GA) for 10 min prior to quenching in 0.1 M glycine–phosphate-buffered saline (PBS). Cells were then permeabilized with 0.2% Triton X-100–PBS (PBS-Triton) and blocked in the same buffer supplemented with 2% bovine serum albumin (BSA) (PBS-Triton-BSA). Cells were incubated with anti-GRA3 (a kind gift from J. F. Dubremetz) (1:20) and anti-GAP45 (23) (1:10,000) primary antibodies diluted in PBS-Triton-BSA for 1 h, followed by PBS-Triton washes (3 times for 5 min each). Cells were incubated with secondary antibodies (Alexa Fluor 488- or Alexa Fluor 594-conjugated goat anti-mouse or goat anti-rabbit IgGs, both diluted 1:1,000) in PBS-Triton-BSA. Nucleus staining was performed by incubating the cells with 4′,6-diamidino-2-phenylindole (DAPI) (50 μg/ml in PBS) prior to the final wash step (3 times for 5 min each). Coverslips were mounted in Fluoromount G (Southern Biotech) on glass slides and stored at 4°C in the dark. Images were acquired on the LSM700 confocal microscope (Zeiss) at the Bioimaging Core Facility of the Faculty of Medicine, University of Geneva. Image processing was done using ImageJ.

**(ix) Size measurement of P. berghei liver stages and analysis of detached cells.** A total of 5 × 10^4^ HeLa cells per well were seeded in 24-well plates and infected the next day with sporozoites. At 48 hpi, the parasite size (area) was determined by density slicing using ImageJ, and infected cells were counted. At 65 hpi, the number of detached cells in the supernatant was counted. The percentage of detached cells was then calculated by dividing the number of detached cells in the supernatant by the number of infected cells at 48 hpi.

**(x) Microscopy of P. berghei liver stage egress.** Late liver stage development and detachment of infected host cells were analyzed by live-cell time-lapse imaging as previously described (13). For this, an LSM880 microscope (airyscan mode) with a Zeiss Plan-Apochromat 63×/1.4 oil objective was used for confocal microscopy. Alternatively, a Leica DM12000B microscope with a Leica Plan-Apochromat 63×/1.2 water immersion objective was used for wide-field microscopy. For multiposition time-lapse imaging, ZEN 2.1 software or Leica Application Suite 2.6 was used. During imaging, cells were kept in a CO_2_ incubator at 37°C. Quantification of PVM rupture in WT and GNAQ-deficient host cells was performed as previously described (14). Image processing was done using ImageJ.

### Data availability.

All data generated or analyzed during this study are included in this article (and the supplemental material).
