# Supplementary figures and images for: CRISPR/Cas9-Based Knockout of GNAQ Reveals Differences in Host Cell Signaling Necessary for Egress of Apicomplexan Parasites
Source: mSphere. 2020 Dec 23;5(6):e01001-20. doi: 10.1128/mSphere.01001-20 (PMC7763550; doi:10.1128/mSphere.01001-20)

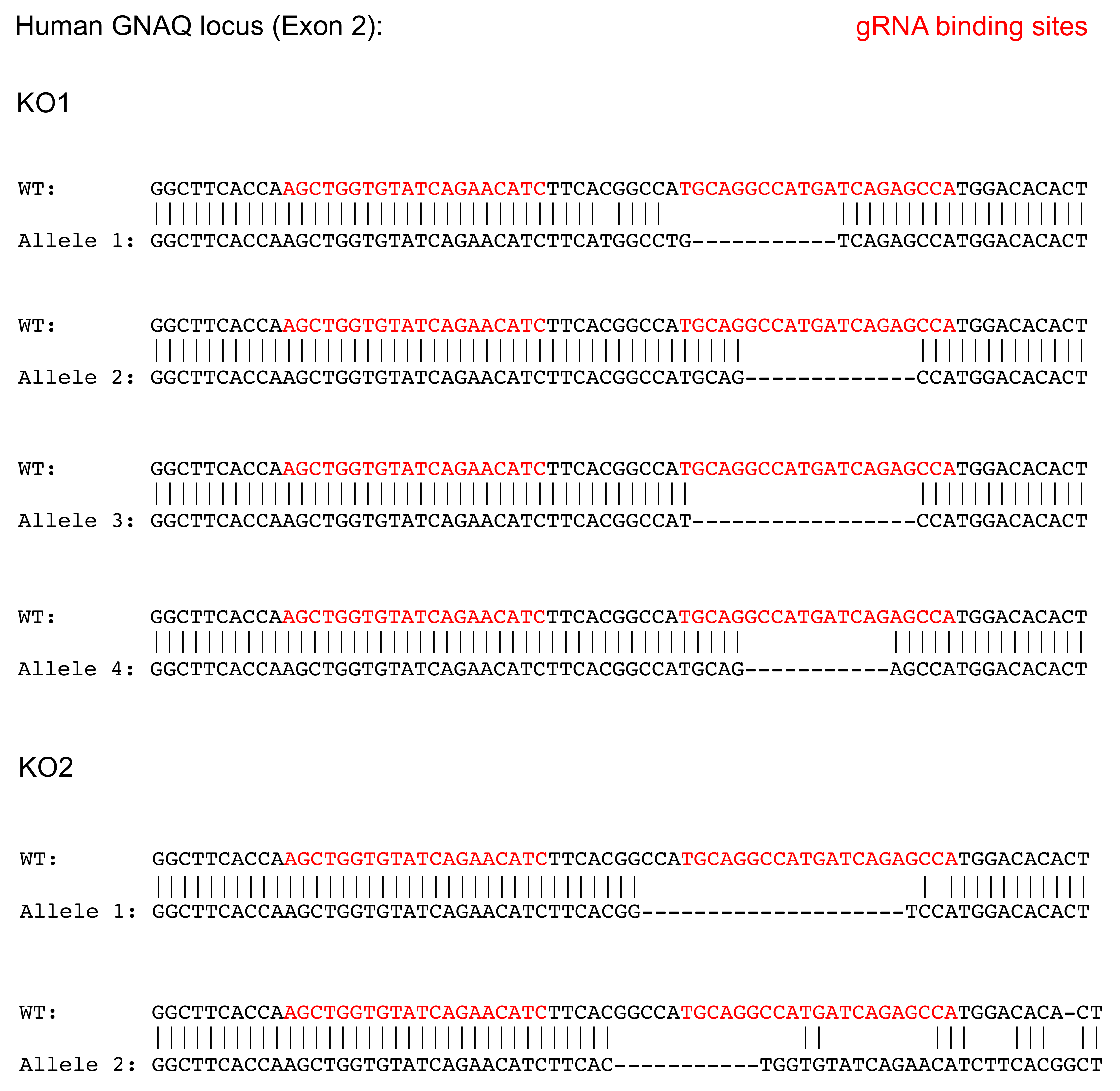

Supplement: FIG S1 [file mSphere.01001-20-sf001.tif]
